# Suprachoroidal implantation of corticosteroid slow release implants for the treatment of cystoid macular edema

**DOI:** 10.1038/s41598-025-05611-y

**Published:** 2025-06-20

**Authors:** Ben Asani, Franziska Kruse, Jakob Siedlecki, Johannes Benedikt Schiefelbein, Benedikt Schworm, Julian Klaas, Tina Herold, Siegfried G. Priglinger

**Affiliations:** Department of Ophthalmology, University Hospital, LMU Munich, Mathildenstrasse 8, 80336 Munich, Germany

**Keywords:** Cystoid macular edema, Implant dislocation, Suprachoroidal implant, Aphakia, Eye diseases, Retinal diseases

## Abstract

In cases of an unstable iris-lens diaphragm, intravitreal corticosteroid slow-release implants (CSRI) may accidentally migrate into the anterior chamber, leading to damage to the corneal endothelium. The purpose of this study was to evaluate the efficacy and safety of a suprachoroidal application of these primarily intravitreal implants to address this issue. In this single-center, retrospective proof of principle study, we observed consecutive cases of patients receiving dexamethasone and fluocinolon acetonide implants that were administered into the suprachoroidal space in patients with CME and instability of the iris-lens diaphragm. Treatment responses of the CME on OCT, incidence of secondary intraocular pressure increases, visual acuity and surgery related complications were evaluated. In total, 16 patients were treated and mean follow up was 1.8 ± 0.97 months. The procedure was well tolerated with no severe intraoperative or postoperative side effects. Mean central retinal thickness (CRT) decreased significantly from 563.13 to 382.12 (p = 0.002). There was no incidence of steroid induced glaucoma. Mean best corrected visual acuity (BCVA) significantly improved from 1.07 logMAR to 0.65 logMAR (p = 0.01). Suprachoroidal implantation of corticosteroid slow-release implants proves to be a safe and feasible alternative for complex eyes with CME and a disruption of the anterior-posterior segment border.

## Background

In cases of cystoid macular edema (CME) — whether postoperative, uveitic, diabetic, or due to vein occlusion — treatment has advanced significantly. While topical non-steroidal anti-inflammatory drugs^1^ and peribulbar injections of triamcinolone^2^ have been used for a long time, the intravitreal injection of corticosteroid slow-release implants (CSRI) (dexamethasone or fluocinolone acetonide) has revolutionized the management of recurrent, severe, and chronic cystoid macular edema, providing long-lasting effects^3–5^. In certain cases, however, a dislocation of the implant into the anterior chamber can lead to (permanent) damage to the corneal endothelium causing severe corneal edema and necessitating a posterior lamellar keratoplasty^6^. Patients with an instable iris-lens diaphragm, such as those with scleral fixated lenses – a technique increasingly popular due to its relatively atraumatic nature (e.g., Yamane-Technique)^7^ - are particularly prone to complications. In these cases, the implant may re-dislocate into the anterior chamber even after successful relocation to the vitreous. Other cases such as large iridotomies or aphakia suffer from similar consequences^6^. To overcome these limitations, suprachoroidal triamcinolone, which has demonstrated high effectiveness in treating uveitic and diabetic macular edema^8–10^, was proposed and is now available as an FDA-approved injection (Xipere^®^, Baush&Lomb, Laval, Canada) in the United States providing a similar safety profile as intravitreal injections with reduced risk of intraocular pressure elevation or accelerated cataract progression^8^. Previous reports have described the option of scleral fixation for dexamethasone or fluocinolone implants as a treatment approach for complex cases of CME^11,12^. However, due to the biodegradable nature of these implants, long-term stability of the scleral fixation may be compromised, potentially resulting in implant migration^11,12^. Furthermore, the scleral fixation procedure is technically more challenging compared to a standard intravitreal injection. Therefore, this study was designed to investigate the safety and efficacy of suprachoroidal implantation of corticosteroid slow-release implants as an alternative approach for the treatment of CME in eyes with disruption of the iris–lens diaphragm.

## Methods

For this interventional case series, we retrospectively and anonymously collected the data of all consecutive patients who underwent a suprachoroidal implantation of a CSRI which was either dexamethasone (Ozurdex^®^, Abbvie, North Chicago, Illinois, USA) or fluocinolone acetonide (Iluvien^®^, Alimera Sciences, Alpharetta, Georgia, USA). For surgery, a 3D heads-up (HUD) system (NGENUITY; Alcon Inc., Fort Worth, Texas, USA; *n* = 45 eyes) which was set up on a standard operating microscope (OPMI Lumera 700 with ReSight; Carl Zeiss Meditec AG; Jena, Germany) was used. All patient data was collected from our institution (University Eye Hospital of the Ludwig-Maximilians University Munich, Germany). Inclusion criteria included chronic cystoid macular edema (uveitic, diabetic or postoperative) impacting visual acuity and contraindication for an intravitreal implant which was defined by a disruption in iris-lens-diaphragm such as aphakia, scleral or iris fixated IOL, large iridectomy and previous dislocation of CSRI. Exclusion criteria included advanced and uncontrolled glaucoma or comorbidities of the sclera such as necrotizing scleritis.

The Ethics Committee of the Medical Faculty of Ludwig-Maximilians-Universität (LMU) Munich confirmed that no formal ethical approval was required for this study (Identifier: 24–0776-KB). The study adhered to the tenets of the Declaration of Helsinki. Written informed consent was obtained from each participant prior to the intervention and all testing outlined herein.

### Suprachoroidal implantation of corticosteroid slow-release implantation

All surgeries were performed by a single very experienced surgeon (S.G.P.) either under retrobulbar block or general anesthesia as an outpatient procedure. The surgical protocol included a radial sclerotomy at the pars-plana whilst leaving the choroid intact, careful separation of the suprachoroidal space with a spatula and injection of dispersive viscoelastic material, sterile release of the suprachoroidal implant (dexamethasone or fluocinolone), careful insertion into the sprachoroidal space and closing of the sclerotomy with one self-absorbing suture (9 − 0 vicryl, Ethicon Inc., Bridgewater, New Jersey, USA).

### Technical considerations for suprachoroidal implantation

1. Sclerotomy Size and Implant Fragility.

A critical factor in successful suprachoroidal implantation is the appropriate sizing of the sclerotomy. If the incision is too small, excessive force may be required to insert the implant, increasing the risk of structural compromise, including fractures or damage to the device.

To mitigate this risk, the sclerotomy should be precisely measured at 2.5–3.0 mm, allowing for smooth and controlled insertion. A self-sealing beveled incision may further facilitate entry while reducing unnecessary resistance. Additionally, gentle and controlled force during implantation is essential to preserve the integrity of the implant and surrounding tissues.

2. Poor Visualization of the Suprachoroidal Space.

Inadequate visualization of the suprachoroidal space can result in unintended transchoroidal placement, with the implant being inadvertently positioned in the vitreous cavity rather than its intended suprachoroidal location.

To optimize surgical precision, adequate illumination and magnification should be employed, utilizing a surgical microscope or loupes for enhanced visibility. Additionally, injecting a small amount of viscoelastic prior to implantation can help delineate the suprachoroidal space, providing a clearer surgical plane and reducing the likelihood of misplacement.

3. Anterior Placement and Choroidal Detachment.

Incorrect anterior positioning of the implant poses a risk of choroidal detachment and potential complications. To prevent this, a posterior approach is recommended, typically 3–4 mm posterior to the limbus. During insertion, the implant should be angled posteriorly to ensure proper placement within the suprachoroidal space and avoid unintended anterior migration.

By adhering to these technical principles, surgeons can enhance procedural accuracy, minimize complications, and improve overall surgical outcomes in suprachoroidal implantation. For experienced surgeons, especially with experience in the suprachoroidal space, there was no significant learning curve. However, given the precision required, initial cases should be performed under supervision or in a controlled learning environment, especially for more inexperienced surgeons.

### Preoperative and postoperative examinations

**Fig. 1:**
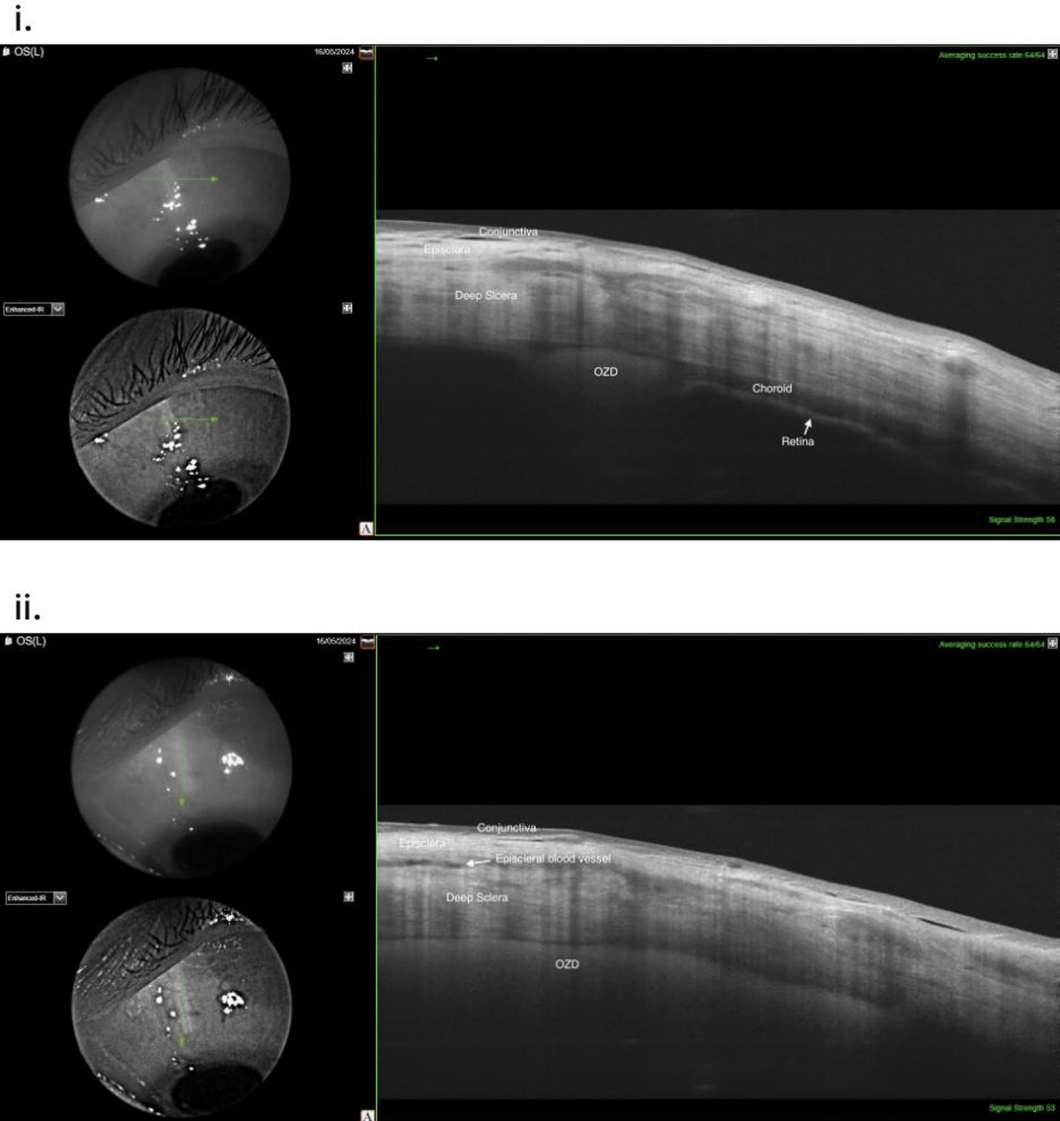
Anterior segment OCT of the sclera using Swept-Source OCT (Dri OCT, Topcon Healthcare, Tokyo, Japan) 3 weeks postoperatively. **i**: *Evidence of correct placement of the implant into the suprachoroidal space at the height of the pars-plana.* **ii**: *Longitudinal scan shows intact suprachoroidal implant.*

Clinical examinations included best-corrected visual acuity testing using standard Early Treatment Diabetic Retinopathy Study (ETDRS) chart at testing distance of 4 m, intraocular pressure measurements using Goldmann applanation tonometry as well as spectral-domain optical coherence tomography of the macula (Spectralis; Heidelberg Engineering GmbH, Heidelberg, Germany). Correct placement and location of the implant was controlled one day and 3 weeks postoperatively with a swept source OCT (DRI OCT, Topcon Corporation, Tokyo, Japan) (Fig. 1).

### Statistical analysis

All statistical analysis was performed using R [R Core Team (2019). R: A language and environment for statistical computing. R Foundation for Statistical Computing, Vienna, Austria; http://www.R-project.org]. Normality of data was assessed using the Shapiro–Wilk Test. We applied the Mann–Whitney-U Test for group comparisons of non-parametric parameters (i.e., X, Y, Z), the independent samples t-test for parametric comparisons (i.e., age) and the Pearson’s chi-squared test for binary comparisons. The level of statistical significance was defined as *p* < 0.05.

### Randomization

Due to the retrospective nature of this study, no randomization was performed. Clinical outcome parameters, including CRT and BCVA, were assessed during routine clinical follow-up by trained ophthalmic personnel who were not masked to the time point of measurement. The intraclass correlation coefficient (ICC) was not calculated, as the measurements were performed by a single examiner per visit and were not reassessed independently.

## Results

This study included 16 eyes of 16 patients treated with dexamethasone (*n* = 14) and fluocinolone implants (*n* = 2). The studies population baseline characteristics are further summarized in Table 1.

**Table 1:** Groups‘ baseline characteristics.

Parameters	Mean ± SD	Range
age	72.3	18–91
female/male ratio	9/7	
IOP (mmHg)	16.4	8–24
CRT (µm)	563.1	310–1010
BCVA preoperatively	1.07	0.4–2.0
lens (pseudophakic/aphakic)	14/2	

12 eyes were diagnosed with postoperative cystoid macular edema, 3 with uveitic cystoid macular edema and one eye was diagnosed with CME after a central retinal vein occlusion. Our cohort did not include patients treated for diabetic macular edema, since there are many intravitreal alternatives with a safe risk and a good effectivity profile such as anti-vascular endothelial growth factor agents. Reasons for a disruption of the iris-lens diaphragm included scleral fixated intraocular lens (*n* = 12), large, open iridotomy (*n* = 1), aphakia (*n* = 2) and one disfigured pupil due to iris coloboma with a history of prior dislocation of intravitreal dexamethasone implants. To have a comparable overview of efficacy, overall follow-up time was similar with an average of 1.8 months. There were no cases lost to follow up. The procedure was very well tolerated, and patients did not present with any serious side effects in this small case series. Some smaller side effects included injection of the conjunctiva, conjunctival hemorrhage, foreign body sensation and one corneal erosion.

The primary anatomical outcome was the change in central retinal thickness (CRT), measured by spectral-domain OCT and its respective software (Heidelberg Eye Explorer Version 1, Heidelberg Engineering, Heidelberg), which is considered a reliable and widely accepted marker for treatment response in macular edema^13,14^.

**Fig. 2:**
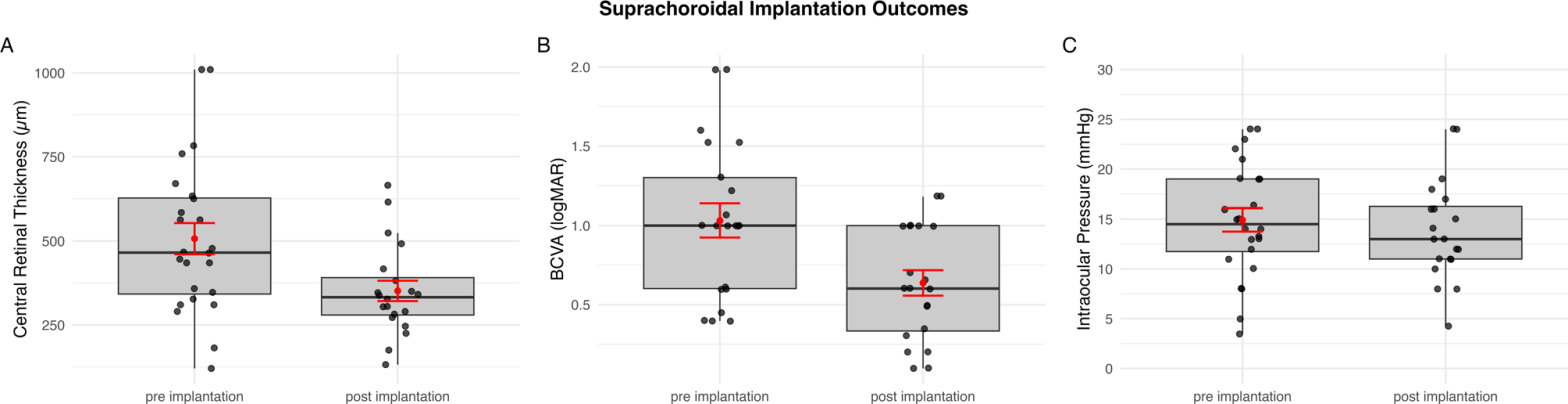
Effectiveness of suprachoroidal dexamethasone implant. *The boxplots show significant improvement in* **(A)** *CRT (563.1 μm pre implantation to 382.1 μm post implantation*,* p-value = 0.002), in ***(B)** *BCVA (1.07 logMar pre implantation*,* 0.65 logMar post implantation*,* p-value = 0.01) and no significant rise in* **(C)** *overall IOP (16.4 mmHg pre implantation to 14.1 mmHg post implantation*,* p-value = 0.15).*

Mean CRT was reduced significantly from 563.1 μm to 382.1 μm (*p* = 0.004). With resolution of macular edema, best corrected visual acuity significantly improved from 1.07 logMAR to 0.65 logMAR (*p* = 0.01). No eye developed steroid-induced glaucoma with IOP > 21 mmHg and on average no significant change in intraocular pressure occurred pre- and postoperatively (from 16.4 mmHg to 14.1 mmHg, *p* = 0.15) (Table 2; Fig. 2). There were no recurrences of CME in the observed time frame.

**Table 2:** Treatment parameters, anatomical and visual outcomes.

Parameter	Pre-Implantation	Post-Implantation	*p*-value
	Mean ± SD	Mean ± SD	
CRT (µm)	563.1	382.1	0.002
*Delta CRT*		*181.0 ± 169.0*	
BCVA (logMar)	1.07	0.65	0.01
IOP (mmHg)	16.4	14.1	0.15

## Discussion

This interventional case series demonstrates the feasibility, safety, and effectiveness of the suprachoroidal implantation of corticosteroid slow-release implants in patients with disruption of the iris-lens diaphragm. In such patients, traditional intravitreal implantation may lead to anterior chamber dislocation, subsequent corneal decompensation, and potentially necessitate lamellar keratoplasty to restore the corneal endothelium. Our three-month results indicate a notable reduction in corneal thickness by 192.79 μm (± 169.3 μm, p-value = 0.004) and an improvement in visual acuity from 1.07 logMar preoperatively to 0.64 logMar postoperatively (p-value = 0.01) thereby improving by 0.43 logMar. Importantly, no cases of steroid-induced glaucoma were observed.

The treatment of cystoid macular edema (CME) or uveitis typically responds well to intravitreal dexamethasone. However, dexamethasone solution has a very short half-life of 5.5 h in the vitreous^15,16^. The advent of slow-release implants, such as Ozurdex^®^, has improved treatment by providing long-lasting effects with a favorable safety profile^4^. Additionally, fluocinolone implants have demonstrated efficacy for up to 36 months^17^.

Alternative methods to traditional implants have been proposed. Peri- or retrobulbar triamcinolone injections, while less potent and with shorter duration compared to slow-release implants, offer a temporary solution. A widely used technique is the Fluocinolone-Loop-Anchoring Technique, which involves scleral fixation of the implant and has shown reproducible effectiveness in treating recalcitrant CME. However, this method can lead to complications such as suture displacement with subsequent implant dislocation while generally requiring a higher level of surgical expertise^11^. Another concern is that degradation of the implant might lead to loosening of the scleral fixation and subsequently to another dislocation of the implant into the vitreous and anterior chamber, especially in the case of the Dexamethasone implant.

Other promising methods include the suprachoroidal injection of triamcinolone acetate, such as the FDA-approved Xipere^®^^8,10^. This method uses a specialized injector (Clearside Medical^®^, ORT, USA) to deliver the substance into the suprachoroidal space. This technique has demonstrated a high safety and efficacy profile, with longer-lasting effects compared to intravitreal triamcinolone and a significantly reduced rate of steroid-induced glaucoma^8^. However, data about its durability are still scarce with reports of efficacy lasting up to three months^8^, and as of to date there is no approval of other official authorities besides the Food and Drug administration (FDA) which clearly puts a limitation on availability for the injector for other markets than the US. Additionally, Dexamethasone is generally more potent than Triamcinolone (up to 6–7 times)^18^, giving the procedure described in this study another possible advantage. An additional benefit of a suprachoroidal application might lie in less steroid induced glaucoma and cataract formation, which was clearly observed in suprachoroidal Triamcinolone where anterior chamber steroid levels were much lower than expected compared to deliverance to the intravitreal space^8^.

Despite the promising results of suprachoroidal injections, there is a lack of substantial publications or case reports on the injection of corticosteroid slow-release implants as of this date except a single case report of suprachoroidal delivery of flucinolone acetonide as described by El Rayes et al.^19^. Concerns regarding the biodegradability of the implants remain. Intravitreally, these implants are fully absorbed within six months^4^. It is unclear whether the same absorption rate occurs in the suprachoroidal space, although it is to be expected. To date, early treated patients have shown no issues related to the absorption and natural degradation of the implant, as evidenced by regular ophthalmological checkups. Swept Source OCT Technology reveals the implant location at the Ora Serrata (See Fig. 2), meaning it does not seem to migrate posteriorly. Additionally, Fluocinolone implants, unlike dexamethasone implants, leave a biocompatible shell in the vitreous after complete absorption. While generally biocompatible with the vitreous, there are no reports on its suprachoroidal tolerability. However, in our study, no significant side effects have been observed up to 10 months postoperatively. Other potential limitations include the retrospective design of this study, lack of masking of outcome assessors to the time points of evaluation, and absence of interobserver reliability testing (ICC). However, the anonymization of patient data reduced the risk of observer bias related to patient identity.

In conclusion, the suprachoroidal implantation of slow-release corticosteroid implants appears to be a safe and effective, innovative method for the treatment of recalcitrant CME in patients with significant disruption of the iris-lens diaphragm. It is a well-tolerated and simple in-office procedure providing the patient with a safe, effective and potentially long-lasting alternative treatment where an intravitreal approach previously failed or seems to be associated with a bigger risk profile. Nonetheless, prospective, randomized double-blinded studies with larger sample sizes are needed to further validate the efficacy and safety of this approach.

## Data Availability

The datasets generated during and/or analysed during the current study are not publicly available due to containing confidential patient information but are available from the corresponding author on reasonable request.
